
JOURNAL INFORMATION
==============================
NLM Title Abbreviation: ScientificWorldJournal
Journal ID: ScientificWorldJournal
NLM Journal ID: 101131163
Journal ID: TSWJ

The Scientific World Journal

ISSN: 2356-6140
EISSN: 1537-744X
Publisher: Wiley

ARTICLE INFORMATION
==============================
PMCID: PMC5974865
PMID: 14646010
DOI: 10.1100/tsw.2003.101
Article ID: 781342
Article version: 1
Subjects: Editorial

Quality of Life Philosophy: When Life Sparkles or Can We Make Wisdom a Science?

Ventegodt Soren 1 *
Andersen Niels JØrgen 2
Merrick Joav 3
The quality of Life Research Center, Teglgårdstræde 4-8, DK-1452 Copenhagen, Denmark
Norwegian School of Management, Sandvika, Norway
National Institute of Child Health and Human Development, Ministry of Social Affairs, Jerusalem, Israel
*Soren Ventegodt: ventegodt@livskvalitet.org
Publication date: 2003
Electronic publication date: 2003 Dec 1
Volume: 3
First page: 1160
Last page: 1163
Copyright: Copyright © 2003 Soren Ventegod et al.
Copyright year: 2003
License: This is an open access article distributed under the Creative Commons Attribution License, which permits unrestricted use, distribution, and reproduction in any medium, provided the original work is properly cited.
License URL: https://creativecommons.org/licenses/by/3.0/

Keywords: quality of life, QOL, philosophy, human development, holistic medicine, public health, Denmark

==============================
Editorial
*TheScientificWorldJOURNAL* (2003) 3, 1160–1163
ISSN 1537-744X; DOI 10.1100/tsw.2003.101

TheScientificWorld
www.thescientificworld.com

### Quality of Life Philosophy: When Life Sparkles or Can We Make Wisdom a Science?

Søren Ventegodt[1,*], Niels Jørgen Andersen[2], and Joav Merrick[3]

*[1]The Quality of Life Research Center, Teglgårdstræde 4-8, DK-1452 Copenhagen K, Denmark; [2]Norwegian School of Management, Sandvika, Norway; [3]National Institute of Child Health and Human Development, Office of the Medical Director, Division for Mental Retardation, Ministry of Social Affairs, Jerusalem and Zusman Child Development Center, Division of Community Health, Ben Gurion University, Beer-Sheva, Israel*

**E-mail**: ventegodt@livskvalitet.org

*Received November 1, 2003; Accepted November 1, 2003; Published December 1, 2003*

---

**KEYWORDS:** quality of life, QOL, philosophy, human development, holistic medicine, public health, Denmark

**DOMAINS:** child health and human development, medical care, behavioral psychology, clinical psychology, nursing

---

### INTRODUCTION

If you take a walk in the woods on a winter morning and see the sun sparkle and glimmer on the newly fallen snow in the treetops, or if you sit one morning on the pier with the one you love and watch the glowing sun rise above the sea, or if you suddenly discover that it has turned dark outside while you have been totally absorbed in your work without a thought for anything else, you may suddenly think that there are far too few moments like these in your life.

Some people experience several high points like these throughout life, when the earth somehow spins the right way just for a brief moment. Others only experience this a few times in their lives. For some, it often happens under certain circumstances, whereas others experience it by pure accident. Sometimes, one of these experiences can change the course of a person's life.

You cannot force these experiences to happen, but invite them by making an effort in your life and doing your utmost in every situation. In order to do so, you need to understand the importance of making the right decision in even the smallest of situations. Self-discipline and personal energy will give you control of your life. When you sincerely do your utmost to be in control of your life, you can catch a glimpse of a major obstacle to success: your inability to understand life, the world around you, and yourself. However, what is missing is not merely intellectual or rational insight. It is primarily existential knowledge, which is related to being genuinely present in life, or in other words, wisdom.

*Corresponding author.
©2003 with author.

### SPARKS OF LIFE

Where does personal energy come from? The tiny sparks that life can hold — like Tinkerbell's magic dust? The abundance and vigor that make us feel utterly alive? The sparks are an expression of what you could call a high personal energy level. Why do some people have so much energy? Why are some people so much more alive? Far more capable of living — better at living their lives and seizing the opportunities in life, solving problems, and so on?

Briefly put, vital energy arises from the experience that life has meaning. The person who knows the meaning of his or her life will persistently fight to realize the dreams and longings that the heart and the soul are full of. The person who, deep within, knows what he or she wants and lives for is strongly, persistently, and continuously struggling to make the dreams and ideals come true.

There are many people who do not strive for quality of life, a meaningful life, but who prefer to play it safe and live a secure, predictable life and cultivate the more superficial pleasures and spontaneous, sensual enjoyments. Typically, such people lead a life that is relaxed or perhaps even lazy. Instead of being in control of their lives, they take things as they come.

When these people experience problems, there is often an increased temptation to put the responsibility for their lives into the hands of experts — physicians, psychologists, social workers, etc. However, in many cases a person may do much more for him- or herself than any expert can. When we begin to invest our resources in our own existence, we can substantially improve our lives over a long period of hard work, often through hard battle and at great pain.

If you already give up at this point and go for the easy life and leave it up to other people to solve your problems, you probably do not value your life all that much after all. Perhaps this irresponsibility reflects your lack of love for life, yourself, your own existence, other people, animals, and plants on earth — love that is a necessary ingredient for a good life.

Naturally it is impossible to supply an exact recipe for a good life, but you could say that each person has to find his or her own way and follow his or her own heart. We believe all people sense the meaning of life in their innermost soul. However, this feeling is not enough. If the meaning of life has to run through your existence like a red thread, it must be articulated in a more deliberate and specific way. You must put it into words. Every single person has to have his own well-reflected and verbally formulated version of the meaning of life. Not until we definitely know what we want will we cease to exist like a dead leaf, always blowing in directions controlled by external powers.

### A SERIES OF PAPERS ON THE PHILOSOPHY BEHIND QUALITY OF LIFE

In the series of papers on quality of life philosophy to appear in this journal (based on work done at the Quality of Life Research Center in Copenhagen, Denmark[1]), we make the argument that knowing the meaning of your own life and living in accordance with it, is the real source of health and well being. We wish to point out that by seizing the meaning of life, you can help yourself experience new health, well being in your personal life, and more talent and ability in your professional life.

With an ever-deeper understanding of life, you can solve more and more difficult problems. By beginning with the simpler problems and ending with the most complicated ones concerning physical and psychological health, we will try to make it plausible to the reader that the art of taking care of your own health is basically about developing a sufficiently profound understanding of life. This also includes adequate ability to solve problems, sufficient self-confidence, and a sufficiently responsible way of living — altogether key ingredients in the quality of life.

Philosophy is not science, it is not made of scientific facts, it is not even based on such facts. It is quite the opposite: Philosophy is the foundation of science, giving it aim, direction, and background. Wisdom is not science. Science has structure, clear concepts, logic, and rules. Wisdom has none of these, because wisdom is about simply being, and therefore knowing. But being and calmness is slowly been substituted by doing and business as the world develops its economical wealth, and likewise is wisdom slowly being substituted by science. To keep wisdom in our human culture in the future, we must try to make a science that contains it, preserves it, and — if possible — even develops it. It is our hope that the science of the global quality of life, developing in medicine these decades and taking its offspring in the brilliant works of Maslow[2] and Antonovsky[3], eventually will contain this wisdom, and that our medical science eventually will be able to remind everybody who needs a better life about the basics of human existence. Papers to appear in the series on philosophy behind quality of life:

- Quality of Life Philosophy I. Quality of Life, Happiness, and Meaning in Life
- Quality of Life Philosophy II. What is a Human Being?
- Quality of Life Philosophy III. Towards a New Biology: Understanding the Biological Connection between Quality of Life, Disease, and Healing
- Quality of Life Philosophy IV. The Brain and Consciousness
- Quality of Life Philosophy V. Seizing the Meaning of Life and Becoming Well Again
- Quality of Life Philosophy VI. The Concepts

### ACKNOWLEDGMENTS

Theses studies was supported by grants from The 1991 Pharmacy Foundation, as well as by supplementary grants from Goodwill-fonden, the JL-Foundation, E. Danielsen and Wife's Foundation, Emmerick Meyer's Trust, the Frimodt-Heineken Foundation, the Hede Nielsen Family Foundation, Petrus Andersens Fond, Wholesaler C.P. Frederiksens Study Trust, Else & Mogens Wedell-Wedellsborg's Foundation and IMK Almene Fond. Our research has been approved by the Copenhagen Scientific Ethical Committee under number (KF)V.100.2123/91.

### REFERENCES

1. Ventegodt, S. (1995) *Quality of Life: Seizing the Meaning of Life and Becoming Well Again.* Forskningcentrets Forlag, Copenhagen. [Danish]
2. Maslow, A. (1962) *Toward a Psychology of Being.* Van Nostrand, Princeton, NJ.
3. Antonovsky, A. (1987) *Unravelling the Mystery of Health. How People Manage Stress and Stay Well.* Jossey-Bass, San Francisco.

**This article should be referenced as follows**:

Ventegodt, S., Anderson, N.J., and Merrick, J. (2003) Quality of life philosophy: when life sparkles or can we make wisdom a science? *TheScientificWorldJOURNAL* **3**, 1160–1163.

### BIOSKETCHES

**Søren Ventegodt, MD,** is the Director of the Quality of Life Research Center in Copenhagen, Denmark. He is also responsible for a Research Clinic for Holistic Medicine in Copenhagen and is a popular speaker throughout Scandinavia. He has published numerous scientific or popular articles and a number of books on holistic medicine, quality of life, and quality of working life. His most important scientific contributions are the comprehensive SEQOL questionnaire, the very short QoL5 questionnaire, the integrated QOL theory, the holistic process theory, the life mission theory, and the Danish Quality of Life Research Survey, 1991–94 in cooperation with the University Hospital of Copenhagen and the late pediatric professor Bengt Zachau-Christiansen. E-mail: ventegodt@livskvalitet.org. Website: www.livskvalitet.org/

**Niels Jørgen Andersen, MSc,** Professor, Department of Innovation and Economic Organization, Norwegian School of Management. This department conducts research and provides teaching in central topics related to innovation, business development, management of global companies, business history, and economic organization. Research activities within the Department are related to four core subjects within the discipline: business history, cooperative organizations, business development and entrepreneurship, and finally studies of industries with a special focus on the electricity industry. He is also the dynamic chairman of the nonprofit organization Stiftelsen Holistisk Medisin Scandinavia, which aims to support the scientific development, research, and documentation of complementary and holistic medicine in Scandinavia. E-mail: niels.j.andersen@bi.no. Website: www.bi.no/users/fgl93013/

**Joav Merrick, MD, DMSc,** is Professor of Child Health and Human Development affiliated with the Zusman Child Development Center and Division of Community Health at the Ben Gurion University, Beer-Sheva, Israel and presently the Medical Director of the Division for Mental Retardation, Ministry of Social Affairs, Jerusalem and the Director of the National Institute of Child Health and Human Development. He has numerous publications in the field of child and human development, rehabilitation, intellectual disability, disability, health, welfare, abuse, advocacy and prevention. Dr. Merrick received the Peter Sabroe Child Award for outstanding work on behalf of Danish Children in 1985 and the International LEGO-Prize ("The Children's Nobel Prize") for an extraordinary contribution towards improvement in child welfare and well being in 1987. E-mail: jmerrick@internet-zahav.net. Website: www.nichd-israel.com
